# Amyloid light-chain amyloidosis presenting as abdominal bloating: a case report

**DOI:** 10.1186/s13256-016-0857-z

**Published:** 2016-03-30

**Authors:** Audry S. Y. Lee, Damian Z. Q. Lee, Farhad F. Vasanwala

**Affiliations:** 1 Department of Internal Medicine, Singapore General Hospital, Outram Road, Singapore, 169608 Singapore; 2 Department of General Medicine, Sengkang General Hospital at Alexandra Hospital, 378 Alexandra Road, Singapore, 159964 Singapore

**Keywords:** Amyloidosis, Gastroparesis, Non-ischemic cardiomyopathy, Weight loss

## Abstract

**Background:**

We present a case of amyloid light-chain amyloidosis with occult plasma cell dyscrasia, with the rare initial presentation of gastroparesis. While amyloidosis is known to affect the gastrointestinal system, rarely do patients present with gastrointestinal symptoms as their first symptom. To the best of our knowledge, this is the first such case reported with a definitive diagnosis made on gastroscopy.

**Case presentation:**

A 52-year-old Malay man with abdominal bloating, early satiety, and weight loss was found to have significant gastroparesis. He had a past medical history of stable non-ischemic cardiomyopathy. Results from initial screening were negative for common causes of gastroparesis, such as diabetes or offending medications. Gastroscopy did not show any mechanical gastric outlet obstruction. Our patient subsequently developed symptoms of postural giddiness, which then prompted further investigations for possible autonomic dysfunction. These finally revealed evidence of systemic involvement, including postural hypotension, speckled myocardium with infiltrative cardiomyopathy on a transthoracic echocardiogram, and multifocal motor neuropathy on nerve conduction studies, from which he had been relatively asymptomatic. These findings were collectively suggestive of infiltrative disease. Retrospective Congo red staining of a gastric biopsy specimen confirmed the diagnosis of gastric amyloidosis. The final diagnosis was amyloid light-chain amyloidosis secondary to plasma cell dyscrasia, which was confirmed by bone marrow examination. Our patients was started on chemotherapy and prokinetic agents, with some improvement in gastrointestinal symptoms on follow-up.

**Conclusion:**

We present this case to highlight that, although rare, gastroparesis can be the initial sole presentation of amyloidosis. It is important for the internist, gastroenterologist, and hematologist to consider amyloidosis as a differential diagnosis in the investigation of gastroparesis and to be vigilant in monitoring for other systemic involvement.

## Background

Delayed gastric emptying and weight loss can be the result of various causes, such as gastroparesis secondary to autonomic dysfunction from diabetes mellitus, or mechanical causes such as ulcers or strictures. We describe a rare case in which the initial presentation was that of gastroparesis and weight loss. Subsequent extensive investigations revealed amyloid light-chain (AL) amyloidosis due to occult plasma cell dyscrasia as the cause of our patient’s symptoms. This diagnosis may explain a previous history of non-ischemic cardiomyopathy.

Gastrointestinal tract involvement is frequently found on postmortem examination in AL amyloidosis, but it causes symptoms in less than 1 % of patients [1]. We present this case to highlight the importance of considering an infiltrative systemic disorder for gastrointestinal symptoms and weight loss. Suspicion of amyloidosis as a cause should be heightened if the patient has prior unexplained organ dysfunction, such as non-ischemic cardiomyopathy in our patient.

## Case presentation

A 52-year-old Malay man was admitted with a 7-month history of abdominal bloating and significant weight loss of 20 kg. He also described reflux symptoms with acid brash, early satiety, and post-meal vomiting, occurring 30–60 minutes after meals.

He had a past medical history of non-ischemic cardiomyopathy diagnosed in a regional hospital in Singapore in 2009. An electrocardiogram performed at that time showed left ventricular hypertrophy with poor R wave progression, with the echocardiogram showing a severely impaired ejection fraction of 15 % and raised pulmonary pressure. He was started on medications for cardiomyopathy but was subsequently lost to follow-up and remained stable without further cardiovascular symptoms. He did not smoke cigarettes nor drink alcohol. A physical examination was unremarkable except for a vague epigastric mass on palpation.

Initial investigations revealed normochromic and normocytic anemia with a hemoglobin level of 11.5 g/dL (normal range, 14–17 g/dL) and albumin of 30 g/L (normal range, 35–55 g/L). Other blood investigations, including urea, creatinine, electrolytes, liver function, thyroid function, autoimmune screen, retroviral screen, and random and fasting blood sugar levels, were normal.

**Fig. 1:**
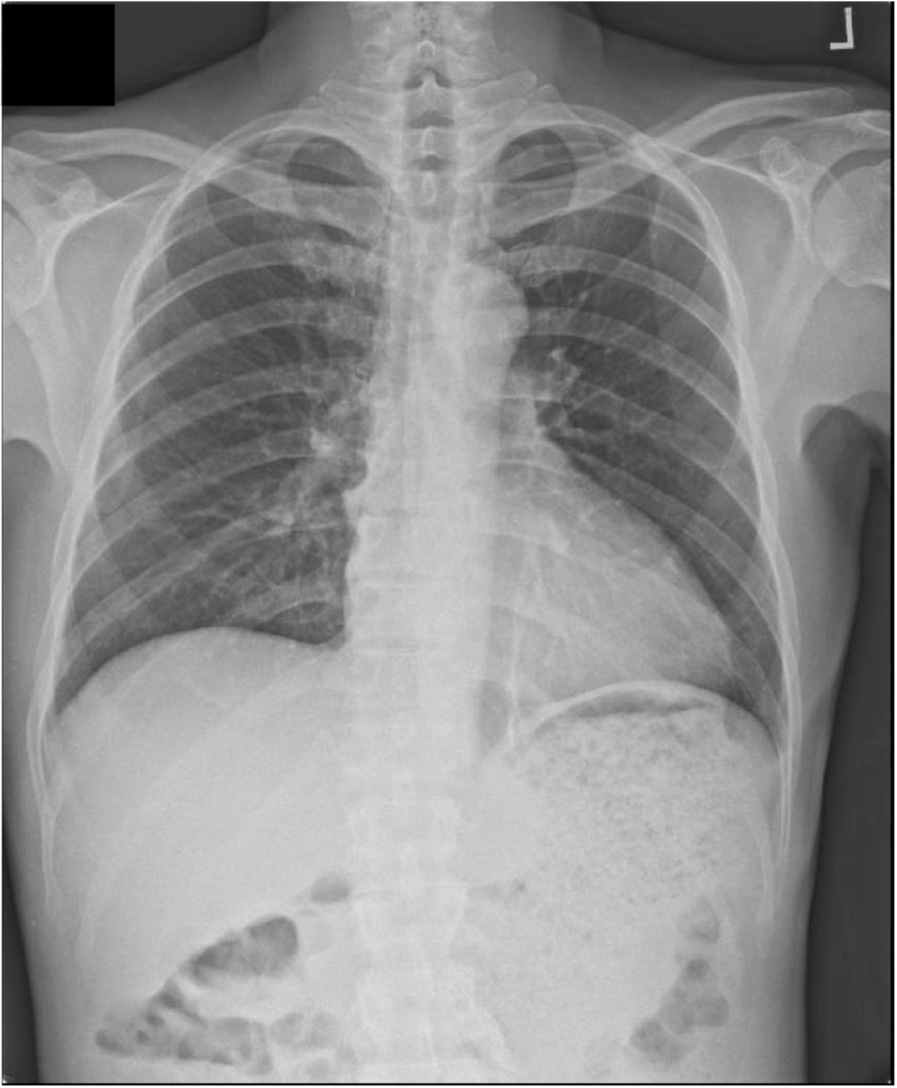
Chest X-ray showing distended gastric outline with food residue

**Fig. 2:**
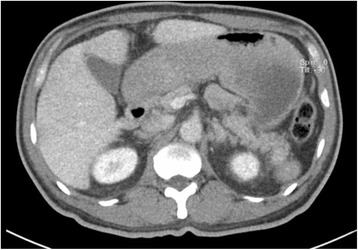
Abdominal computed tomography showing distended stomach with large amounts of gastric content in spite of 4 hours of fasting prior to the scan

**Fig. 3:**
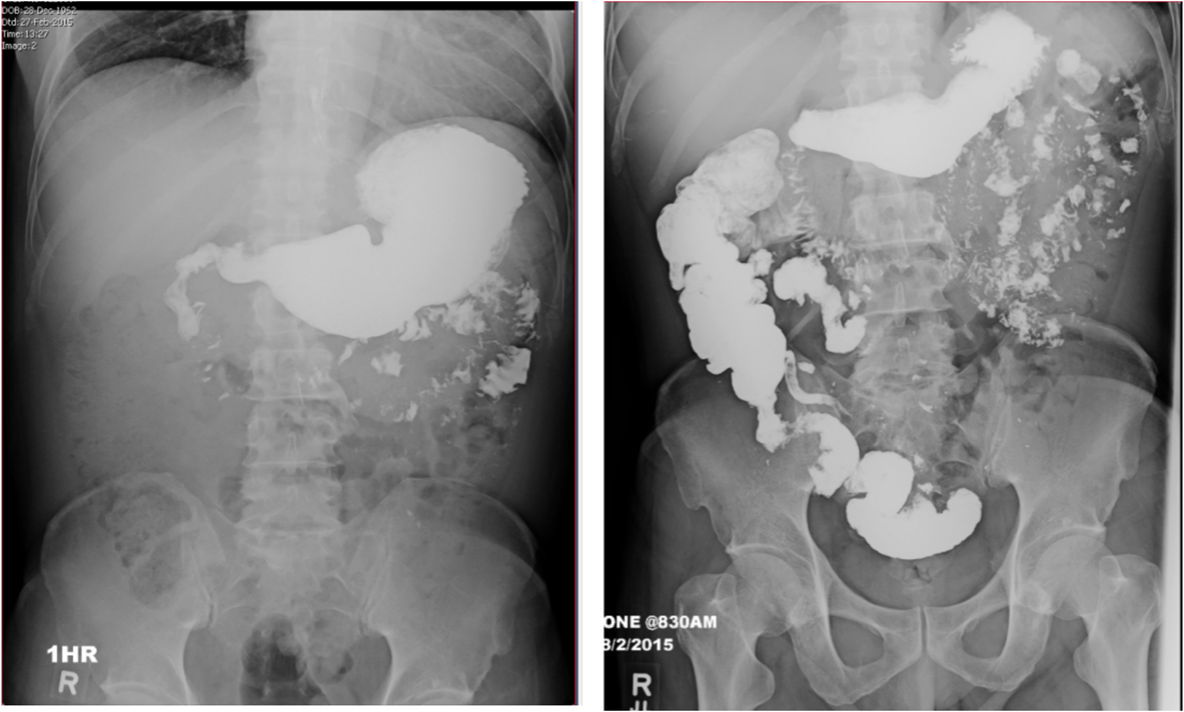
Barium studies showing significant delay in gastric emptying even at 20 hours after barium ingestion, with significant residual gastric barium

A chest X-ray showed an enlarged gastric shadow with evidence of food residue (Fig. 1). A computed tomography scan of his abdomen showed that his stomach was distended with a large amount of gastric content in spite of 4 hours of fasting prior to the scan (Fig. 2). An initial gastroscopy showed reflux oesophagitis and food retention but no evidence of gastric outlet obstruction from ulcers, masses, or strictures. A small bowel series confirmed evidence of delayed gastric emptying with significant residual gastric barium at 20 hours (Fig. 3). Our patient was started on the pro-kinetic agent domperidone with little improvement in symptoms.

Three weeks from his initial presentation to our hospital, our patient complained of postural giddiness. Significant postural hypotension was noted, with his blood pressure falling from 117/75 mmHg when lying to 72/32 mmHg on standing. Further questioning revealed a history of fewer morning erections and possible anhidrosis with little sweating on hot days. He also had mild weakness in his right knee extensor muscles (Medical Research Council grade 4/5), which he attributed to weakness from a fall sustained many years ago. Magnetic resonance imaging of his spine showed non-significant degenerative disc disease and a diffuse low marrow signal on both T1 and T2 sequences, suggestive for marrow replacement or deposition disease.

A repeat gastroscopy was done at a private hospital with a random gastric biopsy showing chronic gastritis. He was readmitted to our hospital for further investigations. It was noted that he had new left-sided periorbital purpura, which had occurred after he had rubbed his eye the day before. Autonomic function studies showed abnormal findings in his lower limbs. Nerve conduction studies showed evidence of left peroneal neuropathy, bilateral carpal tunnel syndrome, and bilateral ulnar neuropathy, attributed to a subclinical multifocal motor neuropathy. Transthoracic echocardiography showed an ejection fraction of 41 %, evidence of biventricular hypertrophy with increased speckling of the ventricular walls, and significant diastolic dysfunction, raising the suspicion of an infiltrative cardiomyopathy.

**Fig. 4:**
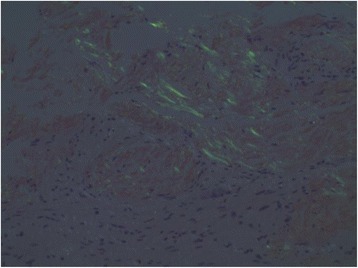
Gastric biopsy showing amorphous material in the submucosa with apple green birefringence on Congo red staining under polarized light

With mounting evidence of a systemic disorder involving autonomic dysfunction with gastroparesis and postural hypotension, infiltrative cardiomyopathy, and multifocal motor neuropathy, a decision was made to investigate further for infiltrative disorders. A repeat histological examination of the previous gastric biopsy was carried out at our hospital with the addition of Congo red staining, which showed apple green birefringence of amorphous material in the submucosa, confirming the diagnosis of amyloidosis (Fig. 4).

Plasma electrophoresis showed polyclonal bands of immunoglobulin G, immunoglobulin M as well as lambda and kappa light chains. Bone marrow aspirate was hypocellular with erythroid and megakaryocytic hyperplasia with moderate plasmacytosis, compatible with plasma cell dyscrasia. Our patient was referred to our Department of Haematology and subsequently started on chemotherapy with bortezomib, cyclophosphamide, and dexamethasone for AL amyloidosis secondary to a plasma cell dyscrasia. On follow-up 2 months later, he had some improvement in his gastrointestinal symptoms with ongoing chemotherapy and use of pro-kinetic agents.

## Discussion

Amyloidosis is a systemic disorder caused by deposition of insoluble abnormal amyloid fibrils that alter the normal function of tissues. The type of protein that is misfolded and the organ or tissue in which they are deposited determine the clinical manifestations of amyloidosis.

The term “amyloid” was first used by Virchow to describe an abnormal extracellular material seen in the liver on autopsy [2]. It was later recognized that this amyloid material uniformly appeared apple green under polarized light with Congo red staining. Electron microscopy showed a common fibrillar nature and beta pleated sheet structure that was key to the pathogenesis of disease.

Various historical classification systems were based on clinical findings or the distribution of organ involvement. The modern classification system of amyloidosis is now based on the type of precursor protein involved [3], made possible by advances in technology that allow for chemical characterization of each protein. The most common forms of amyloidosis are amyloid light-chain (AL or primary systemic), as was the case for our patient; amyloid A (AA or secondary systemic); and familial (abnormal amyloid transthyretin [ATTR]).

The global incidence of systemic amyloidosis is about 8–12 per million persons per year. The initial manifestations can be non-specific, such as weight loss or fatigue. Gastrointestinal symptoms are relatively uncommon, occurring in less than 1 % of patients even though postmortem gastrointestinal involvement can be seen in up to 40 % of cases [4]. If involved, presentation can be with dysphagia [1], gastroparesis, pseudo-obstruction [5], malabsorption [6], or even bowel ischemia or infarction [7] due to amyloid deposition in the intestinal vascular supply. As in our patient’s case, it is rare for a patient to present initially with such overt gastrointestinal symptoms.

Definitive diagnosis of amyloidosis depends on a biopsy of the affected tissue or organ, or of rectal or subcutaneous fat in systemic disease. Treatment of systemic amyloidosis remains unsatisfactory, with a median prognosis of 13 months [8]. Treatment in AL amyloidosis is based on a regime of chemotherapy and stem cell transplantation if possible. Supportive measures appropriate to the organ systems affected can be initiated to improve quality of life.

## Conclusion

This case has highlighted that amyloidosis can initially present with predominantly gastrointestinal symptoms alone, with other clues to systemic involvement occurring much later. Although a rare cause of gastroparesis and weight loss, internists, gastroenterologists, and hematologists should consider amyloidosis as an important differential diagnosis, and should be vigilant in monitoring for later developments indicating other systemic involvement. A high index of suspicion is required to consider amyloidosis when other more common causes such as autonomic dysfunction due to diabetes, malignancy, or mechanical gastric outlet obstructions have been excluded.

## Consent

Written informed consent was obtained from the patient for publication of this case report and any accompanying images. A copy of the written consent is available for review by the Editor-in-Chief of this journal.
